# Supplementary material for: Past and Recent Effects of Livestock Activity on the Genetic Diversity and Population Structure of Native Guanaco Populations of Arid Patagonia
Source: Animals (Basel). 2021 Apr 23;11(5):1218. doi: 10.3390/ani11051218 (PMC8146674; doi:10.3390/ani11051218)
Supplement: Supplementary file 1 [file animals-11-01218-s001.zip › Supplementary Material S2 24.2.21 en formato-2.docx]

**Supplementary Material**

**S.1.Supplementary Table 1***.* *Lama guanicoe* samples from southern Monte and northern Patagonian Steppe of Argentina. The type of sample, geographic position, localities population, death cause, ontogeny stage and sex are indicated for each sample (See excel file: Supplementary Table 1.xlsx).

**S.2. Supplementary Table 2***.* Alignment of polymorphic sites for 82 north Patagonian guanaco haplotypes obtained for 514bp of Control Region sequences. For each haplotype, locabilities (LB = Loma Blanca; MS = Meseta Somuncura; TE = Telsen; PV = Península Valdés; BD = Bajada del Diablo; LP = Las Plumas; AM = Ameghino) and the frequency of each haplotype are detailed.

| Haplotype | 2 | 4  3 | 4  4 | 6  9 | 8  4 | 1  7  2 | 1  8  0 | 1  9  1 | 2  1  8 | 2  3  4 | 2  7  0 | 2  9  2 | Population | | | | | | | N |
| --- | --- | --- | --- | --- | --- | --- | --- | --- | --- | --- | --- | --- | --- | --- | --- | --- | --- | --- | --- | --- |
|  |  |  |  |  |  |  |  |  |  |  |  |  | LB | MS | TE | PV | BD | LP | AM | Total=82 |
| 1 | T | G | A | C | A | A | T | G | T | C | T | T | 14 | 9 | 6 | 7 | 1 | 2 | 9 | 48 |
| 2 | . | . | . | . | . | . | . | A | . | . | . | . | 3 | 2 | 3 |  |  | 1 | 2 | 11 |
| 3 | . | . | T | . | . | . | . | . | . | . | . | . | 1 |  | 2 | 2 | 2 |  |  | 7 |
| 4 | . | . | . | . | . | . | . | . | C | . | . | . | 2 |  |  |  |  |  |  | 2 |
| 5 | . | . | . | T | . | . | . | . | . | . | . | . |  | 2 |  | 2 |  |  |  | 4 |
| 6 | . | . | . | . | . | . | . | . | . | T | . | . |  | 1 |  |  |  |  |  | 1 |
| 7 | . | A | . | . | . | . | . | . | . | T | . | . |  |  | 1 |  |  |  |  | 1 |
| 8 | . | . | . | . | . | . | . | . | . | . | C | . |  |  | 1 |  |  |  |  | 1 |
| 9 | . | . | . | . | G | . | . | . | . | . | . | . |  |  |  | 1 |  |  |  | 1 |
| 10 | . | . | T | . | . | . | . | . | . | . | . | . |  |  |  | 1 |  |  |  | 1 |
| 11 | . | . | . | . | . | . | . | . | . | . | . | C |  |  |  |  | 1 |  |  | 1 |
| 12 | C | . | T | . | . | . | . | . | . | . | . | . |  |  |  |  |  | 1 |  | 1 |
| 13 | . | . | T | . | . | G | . | . | . | . | . | . |  |  |  |  |  | 1 |  | 1 |
| 14 | . | . | . | . | . | . | C | . | . | . | C | . |  |  |  |  |  |  | 1 | 1 |
| 15 | C | . | T | . | . | . | . | . | . | . | . | . |  |  |  |  |  |  | 1 | 1 |

**Supplementary Table 3.** FST with a Bonferoni correction. Fst values are listed below the diagonal and p-values are above the diagonal ( signicance level p-value ≤ 0.002). Localities names are: LB = Loma Blanca; MS = Meseta Somuncura; TE = Telsen; PV = Península Valdés; BD = Bajada del Diablo; LP = Las Plumas; AM = Ameghino.

|  | Group 1 LB | Group 2 | MS | PV | TE | Group 3 | BD | LP | AM |
| --- | --- | --- | --- | --- | --- | --- | --- | --- | --- |
| Group 1 (LB) | - | **0** | **0** | **0** | **0** | **0** | **0,001** | **0** | **0** |
| Group 2 | **0.09145** | - |  |  |  |  |  |  |  |
| MS | **0,08026** |  | - | 0,02188 | 0,52223 |  | 0,1089 | **0,0002** | 0,07079 |
| PV | **0,11542** |  | 0,01054 | - | 0,03069 |  | 0,00356 | 0 | **0,0003** |
| TE | **0,08308** |  | -0,00345 | 0,01047 | - |  | 0,01426 | 0 | 0,00614 |
| Group 3 | **0.09820** | **0.02506** |  |  |  | - |  |  |  |
| BD | **0,11554** |  | 0,0347 | 0,04613 | 0,0442 |  | - | 0,03534 | 0,09494 |
| LP | **0,12356** |  | **0,03179** | 0,05452 | 0,04956 |  | 0,05393 | - | 0,05 |
| AM | **0,07968** |  | 0,01081 | **0,03981** | 0,02297 |  | 0,03798 | 0,01899 | - |

**Supplementary Table 4.** Estimation of Migration (SD) using BAYESASS. Head of columns indicate the source of animal by site, and head of files indicate destination. Values > 0.1 are in bold. Localities names are: LB = Loma Blanca; MS = Meseta Somuncura; TE = Telsen; PV = Península Valdés; BD = Bajada del Diablo; LP = Las Plumas; AM = Ameghino.

|  | LB | MS | PV | TE | BD | LP | AM |
| --- | --- | --- | --- | --- | --- | --- | --- |
| LB | - | 0.0127(0.0123) | 0.0125(0.0120) | 0.0127(0.0122) | 0.0132(0.0127) | 0.0182(0.0164) | 0.0127(0.0121) |
| MS | 0.0152(0.0145) | - | **0.2419(0.0312)** | 0.0151(0.0144) | 0.0152(0.0144) | 0.0157(0.0153) | 0.0152(0.0148) |
| PV | 0.0155(0.0149) | 0.0151(0.0144) | - | 0.0153(0.0145) | 0.0153(0.0146) | 0.0193(0.0179) | 0.0154(0.0147) |
| TE | 0.0151(0.0145) | 0.0151(0.0143) | **0.2407(0.0317)** | - | 0.0149(0.0143) | 0.0175(0.0162) | 0.0149(0.0144) |
| BD | 0.0300(0.0271) | 0.0305(0.0274) | 0.0309(0.0276) | 0.0302(0.0274) | - | **0.1515(0.0477)** | 0.0309(0.0278) |
| LP | 0.0161(0.0151) | 0.0159(0.0152) | 0.0214(0.0195) | 0.0161(0.0151) | 0.0160(0.0152) | - | 0.0159(0.0153) |
| AM | 0.0134(0.0129) | 0.0159(0.0153) | 0.0174(0.0158) | 0.0162(0.0154) | 0.0159(0.0155) | **0.2234(0.0316)** | - |

**Supplementary Table 5.** Prior distributions of the demographic parameters inferred using MsVar, including the estimated current effective population size (No), the ancestral effective population size (Nt), the mutation rate (mu), and the time of the bottleneck in years (t). The prior distributions were log-normal distributions parameterized with the mean and standard deviation (SD) for each parameter and truncated at zero following Storz and Beaumont (2002). The values on the table correspond to the priors of each of the parameters. LB = low boundary (mean – SD), HB = high boundary (mean +SD). The hyperprior mean of means and variance of means had the same values as the mean and range in the prior, respectively, and the mean of variances and variance of variances were left as default.

|  | No | | | Nt | | | mu | | | t | | |
| --- | --- | --- | --- | --- | --- | --- | --- | --- | --- | --- | --- | --- |
| Scenario | LB | mean | HB | LB | mean | HB | LB | mean | HB | LB | mean | HB |
| Bottleneck | 10 | 100 | 1,000 | 500 | 5,000 | 50,000 | 0.000032 | 0.00032 | 0.0032 | 10 | 100 | 1,000 |
| Stable | 50 | 500 | 5,000 | 50 | 500 | 5,000 | 0.000032 | 0.00032 | 0.0032 | 100 | 1,000 | 10,000 |
| Expansion | 100 | 1,000 | 10,000 | 31 | 316 | 3,162 | 0.000032 | 0.00032 | 0.0032 | 40 | 400 | 4,000 |

**
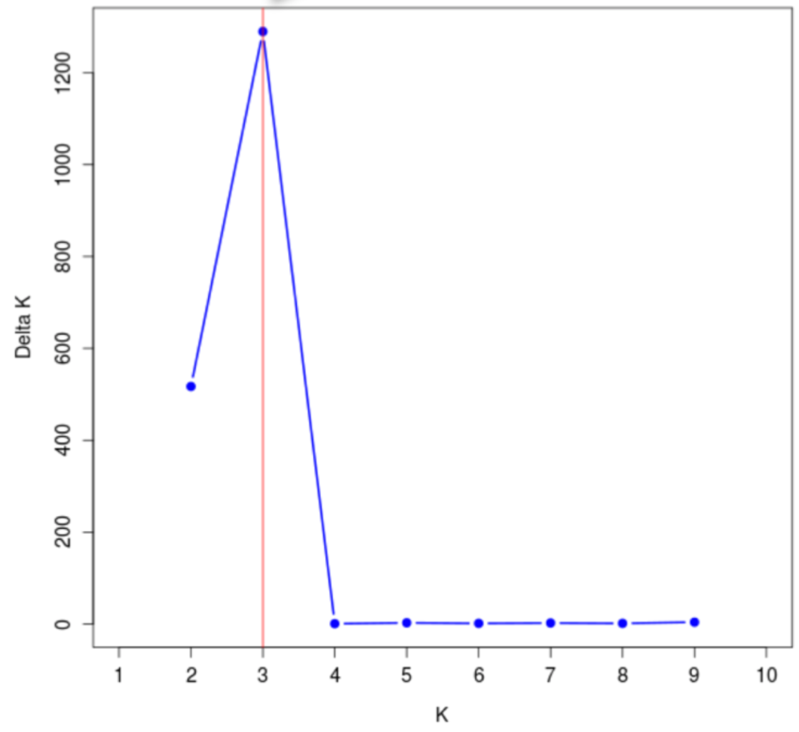
**

**Supplementary Figure 1.** Relative support for the number of guanaco populations based on the microsatellite data set. Δ*K* (Delta K = mean (∣L’’(K)∣)/SD(L(K))) following Evanno et al. (2005) as a function of K.

**
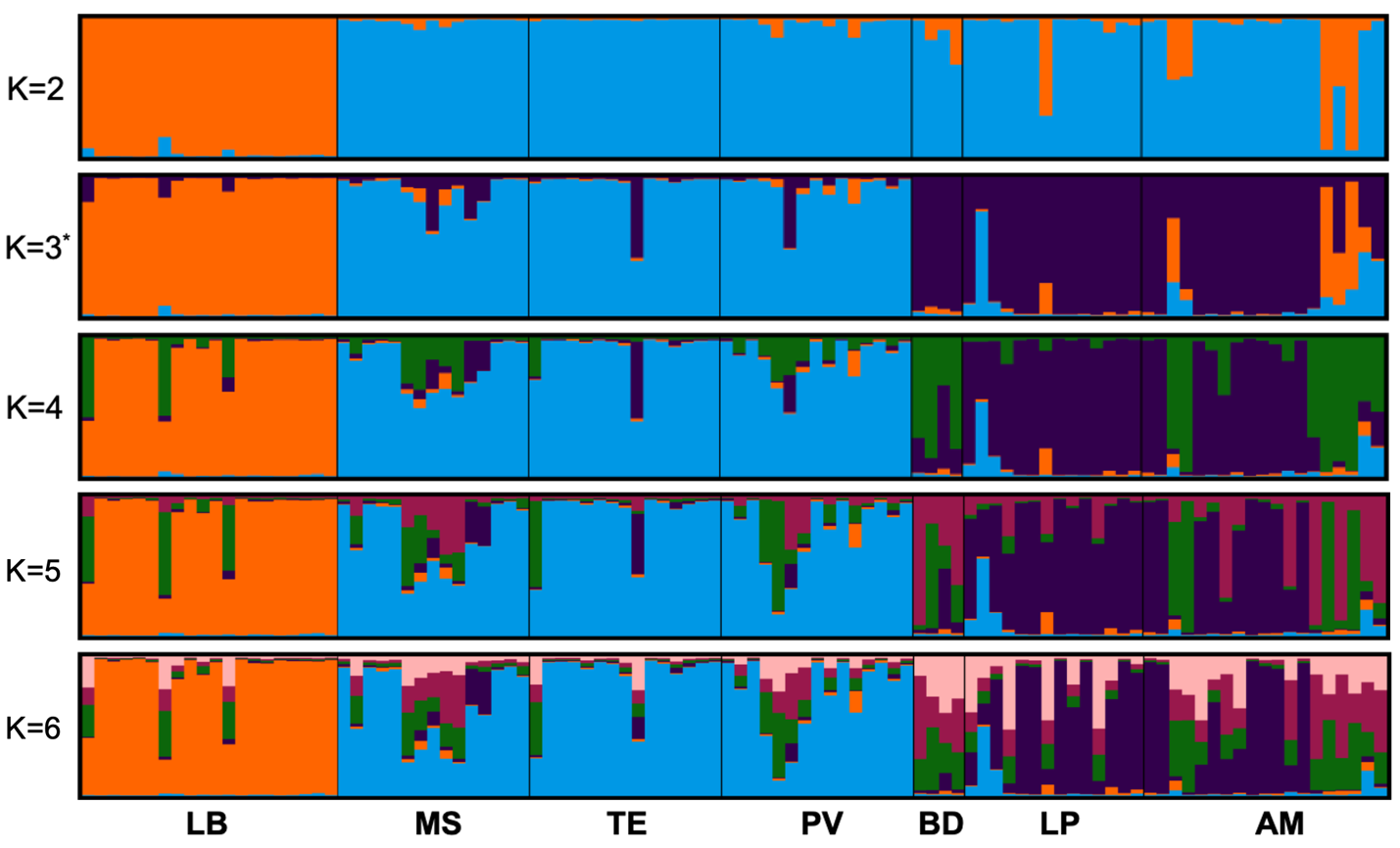
**

**Supplementary Figure 2**. Structure analysis results of 102 guanacos *Lama guanicoe* from seven localities in in Chubut and Rio Negro Provinces in the Patagonia of Argentina based on five 5 repetitions of K1 through K10 with 1,000,000 repetitions and a burn-in of 20,000 and a model assuming correlation between admixture and allele frequency. Localities names are: LB = Loma Blanca; MS = Meseta Somuncura; TE = Telsen; PV = Península Valdés; BD = Bajada del Diablo; LP = Las Plumas; AM = Ameghino.


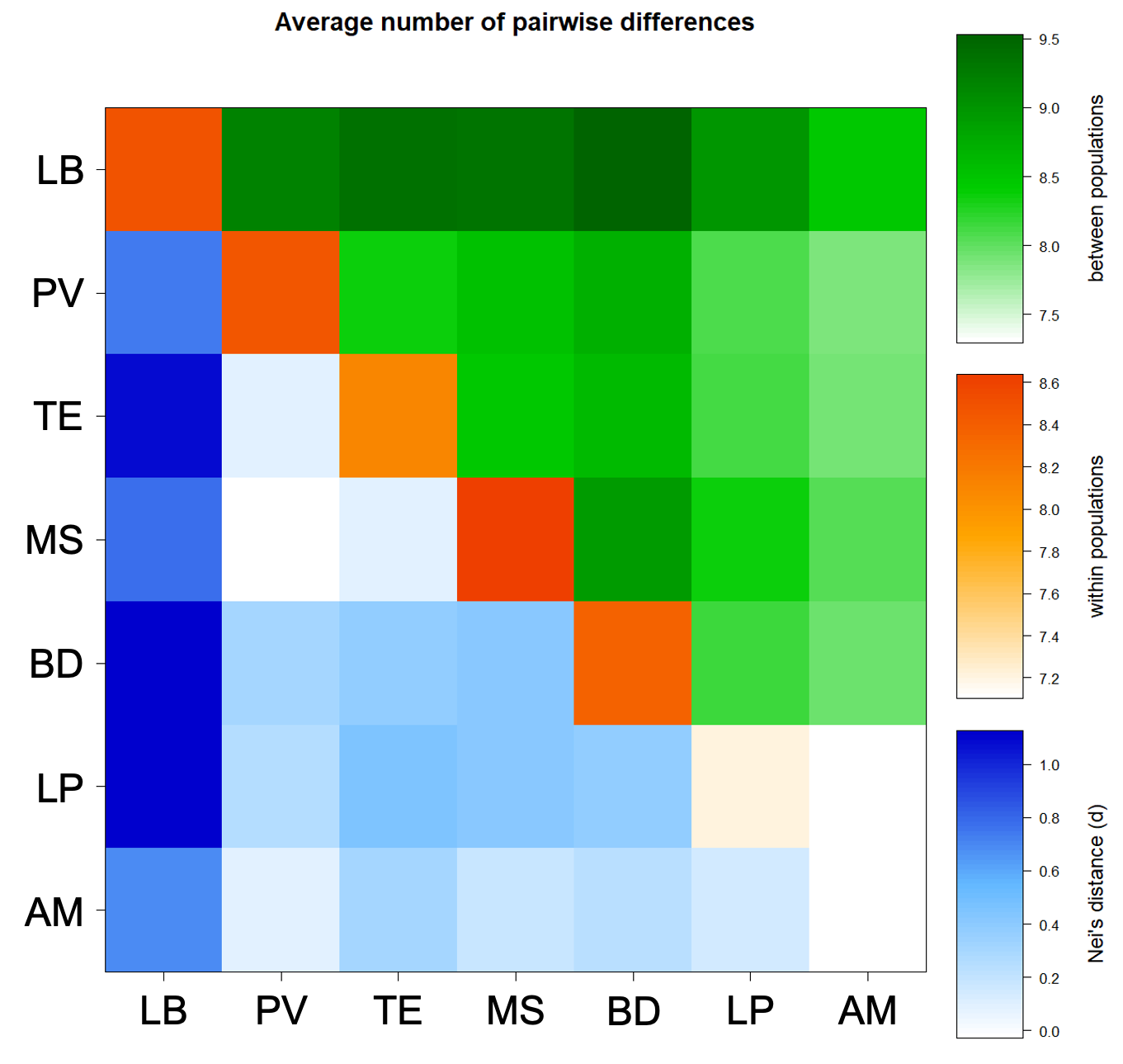


**Supplementary Figure 3.** Plot of the pairwise differences showing the comparison between locality groups. The plot includes the average number of pairwise differences between populations (green), the average number of pairwise differences within populations (orange) and the corrected average pairwise difference (blue). Localities names are: LB = Loma Blanca; MS = Meseta Somuncura; TE = Telsen; PV = Península Valdés; BD = Bajada del Diablo; LP = Las Plumas; AM = Ameghino.


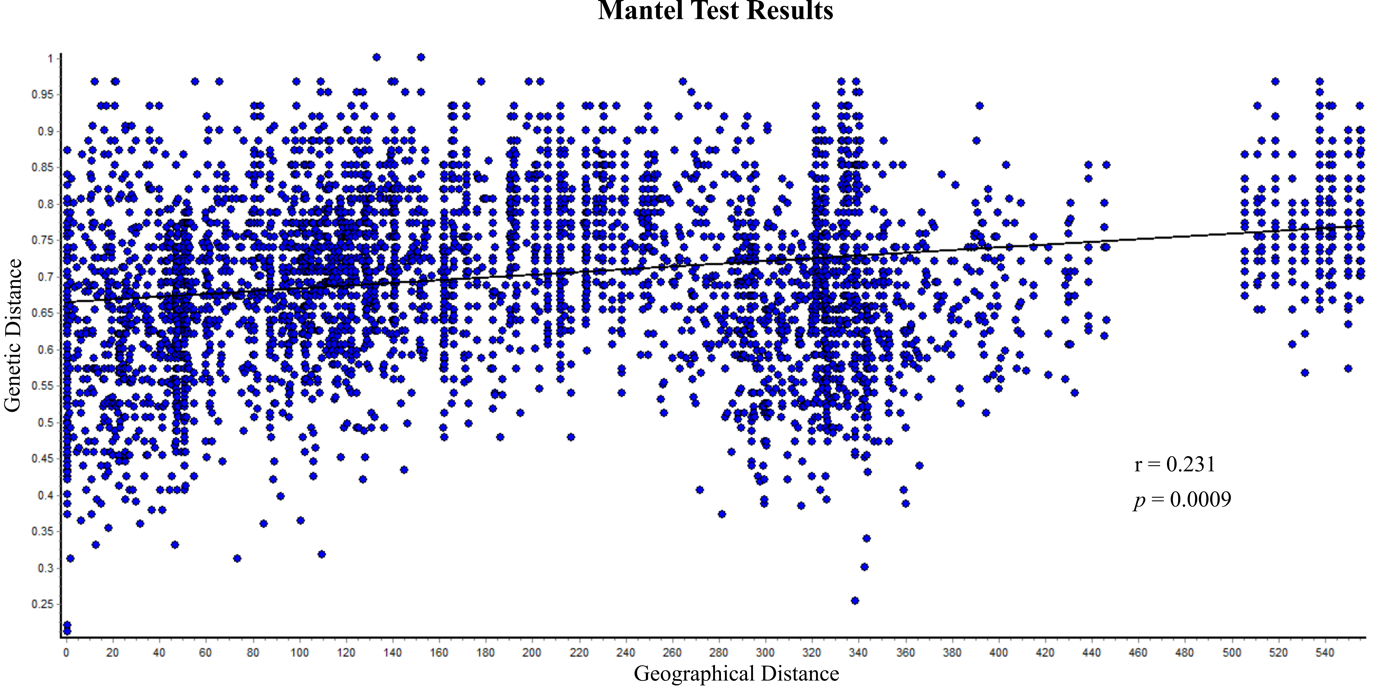


**Supplementary Figure 4** Mantel test graphic showing the results of the isolation by distance (IBD) analysis in ALLELES IN SPACE (Miller, 2005). r = 0.231, p < 0.0009.


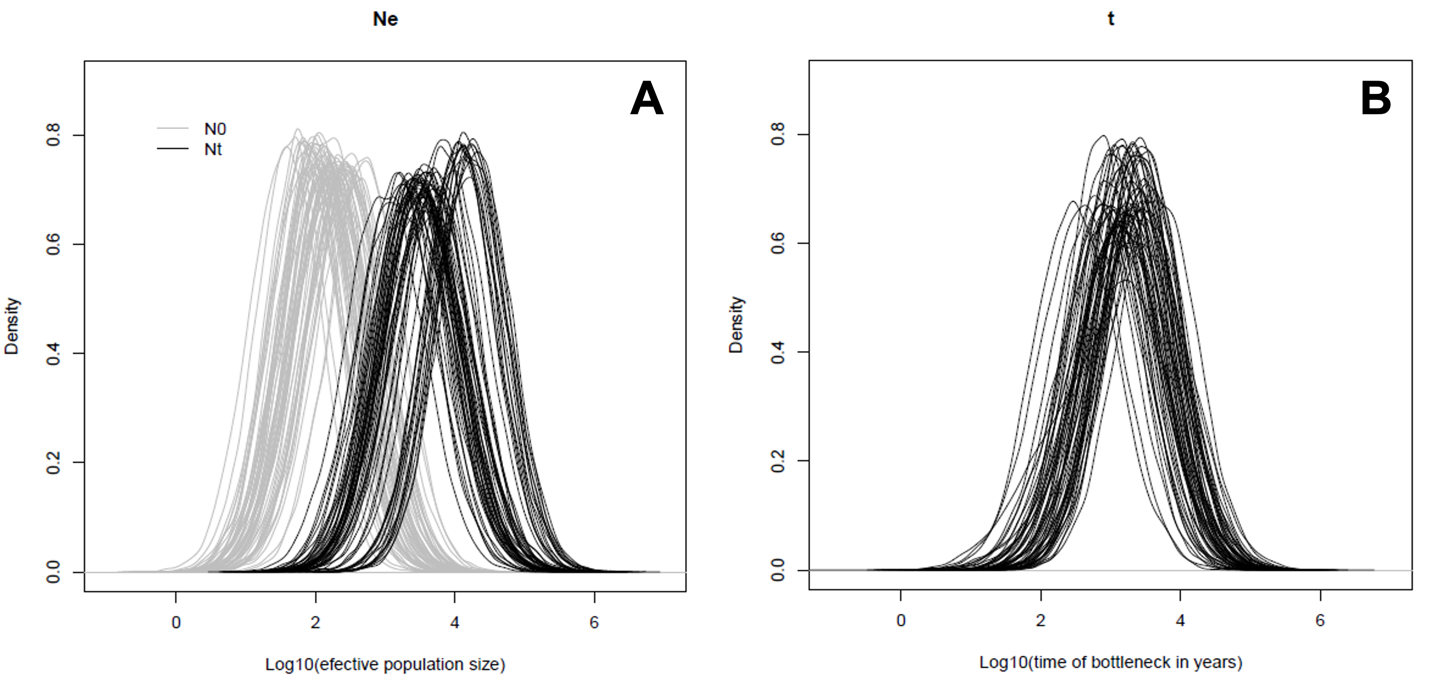


**Supplementary Figure 5**. Demographic analysis of guanaco (*Lama guanicoe*) populations using MsVar demographic models assuming population expansion, no demographic change and a bottleneck. **A** depicts the posterior distributions of the present effective population size (N0) and the ancestral effective population size (Nt) and **B** shows the estimated time when the demographic event occurred.
